# A protein with simultaneous capsid scaffolding and dsRNA-binding activities enhances the birnavirus capsid mechanical stability

**DOI:** 10.1038/srep13486

**Published:** 2015-09-04

**Authors:** Johann Mertens, Santiago Casado, Carlos P. Mata, Mercedes Hernando-Pérez, Pedro J. de Pablo, José L. Carrascosa, José R. Castón

**Affiliations:** 1 Instituto Madrileño de Estudios Avanzados en Nanociencia (IMDEA Nanociencia) Campus Cantoblanco, 28049 Madrid, Spain; 2 Unidad Asociada de Nanobiotecnología CNB-CSIC-IMDEA, Cantoblanco, E-28049 Madrid, Spain; 3 Department of Structure of Macromolecules, Centro Nacional de Biotecnología/CSIC, Campus Cantoblanco, 28049 Madrid, Spain; 4 Departamento de Física de la Materia Condensada C03, Universidad Autónoma de Madrid, 28049 Madrid, Spain

## Abstract

Viral capsids are metastable structures that perform many essential processes; they also act as robust cages during the extracellular phase. Viruses can use multifunctional proteins to optimize resources (e.g., VP3 in avian infectious bursal disease virus, IBDV). The IBDV genome is organized as ribonucleoproteins (RNP) of dsRNA with VP3, which also acts as a scaffold during capsid assembly. We characterized mechanical properties of IBDV populations with different RNP content (ranging from none to four RNP). The IBDV population with the greatest RNP number (and best fitness) showed greatest capsid rigidity. When bound to dsRNA, VP3 reinforces virus stiffness. These contacts involve interactions with capsid structural subunits that differ from the initial interactions during capsid assembly. Our results suggest that RNP dimers are the basic stabilization units of the virion, provide better understanding of multifunctional proteins, and highlight the duality of RNP as capsid-stabilizing and genetic information platforms.

A capsid forms the interface between a viral genome and its greatly varied environment, from the relatively mild conditions within a cell host to potentially extreme changes in its external milieu^1^. Capsids are not mere genome containers, however, but participate in many functions, including virus maturation, transport within the host, receptor recognition, and uncoating (controlled disassembly) for genome release; some capsids also have an active role in viral replication^2^,^3^,^4^. This functional diversity throughout the viral cycle makes capsids an excellent model for analyzing the dynamics of macromolecular assemblies, and they might also serve as nanoplatforms in materials science applications. In addition to X-ray crystallography and three-dimensional cryo-electron microscopy studies of the capsid, atomic force microscopy (AFM) is a complementary tool for examining the close relationship between physical properties and biological function. AFM provides physicochemical information not evident from structural data, such as direct measurement of the mechanical rigidity and breaking force of the viral capsid in physiological conditions^5^,^6^,^7^.

We used AFM to analyze the biophysical properties of a non-enveloped icosahedral virus. Infectious bursal disease virus (IBDV) is an avian double-stranded (ds)RNA virus of the Birnaviridae family, which encompasses functional and structural features of positive and negative single-stranded (ss)RNA viruses^8^. IBDV has a polyploid bipartite dsRNA genome (segments A and B of 3.2 and 2.8 kbp, respectively) enclosed within a single-layered ~70 nm-diameter capsid with T = 13 *l* geometry^9^,^10^. The two dsRNA segments are assembled into ribonucleoprotein particles (RNP) containing numerous nucleoprotein monomers^11^,^12^.

RNP-A has two open reading frames (ORF). The short ORF encodes VP5, a nonstructural protein involved in virus egress, and the large ORF codes for a polyprotein that is cotranslationally processed by the VP4 viral protease^13^ to yield the precursor capsid protein pVP2 as well as VP3 and VP4^14^,^15^. pVP2 can be further processed by a host protease^16^ and/or by VP2 itself^17^ to yield mature VP2 and several C-terminal fragments. RNP-B is monocistronic and encodes the RNA-dependent RNA polymerase (RdRp) VP1, which is packaged within the virion as a free protein or is covalently linked to the 5′ ends of the dsRNA molecules (the so-called VPg).

VP3 (258 residues) is a multifunctional protein that, in addition to its RNA-binding activity, interacts with itself^18^,^19^, with pVP2^20^,^21^, or with VP1^22^,^23^,^24^. VP3 acts as a scaffolding protein during capsid morphogenesis and, through electrostatic interactions of its final five, mainly acidic residues, co-assembles with pVP2^20^. During capsid assembly, VP3 recruits VP1 molecules via its 16 C-terminal-most residues, and also encapsidates the viral genome. In virus replication, the VP3 C terminus functions as an RdRp transcriptional activator^23^ and as a suppressor of the RNA silencing machinery^25^. The X-ray structure shows VP3 organization as a dimer in its central region^18^. Regions responsible for some of these varied roles map on overlapping C-terminal segments that are intrinsically unstructured (as deduced from X-ray analysis), suggesting that VP3 is a moonlighting protein^26^,^27^, similar to paramyxovirus nucleoproteins^28^.

In IBDV-infected cells, the assembly pathway gives rise mainly to virions that package four RNP, although minor discrete viral populations with lower RNP content are also formed. In our experiments, we correlated greater virus rigidity with greater RNP copy number inside the viral capsid and thus, with IBDV populations with the highest infectivity rates. AFM mechanical fatigue experiments at low force indicated that RNP organized as dimers within the capsid, which increased viral particle stability in the harsher extracellular environment. Our results indicate once VP3 is bound to the viral genome, it mediates capsid stability through a new set of interactions with the mature VP2 protein. These analyses support simultaneous multifunctionality as a mode of VP3 action. Identification of these physical features using a relatively simple viral system susceptible to manipulation indicates new approaches to the study of moonlighting proteins.

## Results

### Elastic response of IBDV populations

At least six major IBDV populations of extracellular viral particles (termed E1-E6) can be purified from IBDV-infected avian cells by ultracentrifugation on a CsCl density gradient (Supplementary Fig. 1a). Whereas E2-E6 have similar protein composition and are T = 13 capsids, E1 is comprised of empty T = 1 and T = 13 capsids (Supplementary Fig. 1b). E2-E6 capsids have constant amounts of pVP2/VP2, VP3 and VP1 (780, ~450 and ~12 copies per capsid, respectively^8^). Their genomic content nonetheless differs, with 1, 2, 3 and 4 dsRNA segments (A or B), respectively, in E2, E3, E4 and E5-E6 populations^8^. The molecular differences remain to be established between E5 and E6 particles, whose infectivity and sedimentation behavior differ slightly. Differences in amounts of single-stranded oligonucleotides, which form up to 25% of RNA in purified reovirus, could explain the higher sedimentation coefficient for the E6 population^29^. Alternatively, E5 and E6 particles may have distinct properties, for example in capsid permeability to cesium. IBDV polyploidy is an evolutionarily advantageous mechanism that generates fully infectious virus particles with high probability.

**Figure 1:**
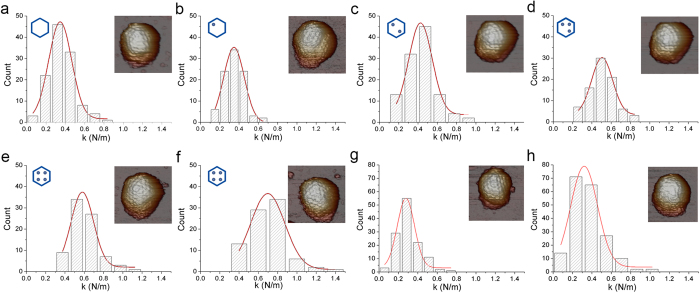
Mechanical rigidity of IBDV E1-E6 populations and IBDV T = 13 VLP. (**a–f**) Histogram of slopes of the indentation curves carried out for (**a**) E1, (**b**) E2, (**c**) E3, (**d**) E4, (**e**) E5 and (**f**) E6 IBDV capsids. They show the rigidity values (spring constant, k) for individual particles after nanoindentation. The k value for each population was calculated by Gaussian fits, (**a**) k_E1_ = 0.346 ± 0.118 N/m; (**b**) k_E2_ = 0.347 ± 0.106 N/m; (**c**) K_E3_ = 0.426 ± 0.121 N/m; (**d**) k_E4_ = 0.505 ± 0.109 N/m; (**e**) k_E5_ = 0.580 ± 0.116 N/m; and (**f**) k_E6_ = 0.697 ± 0.177 N/m. AFM images (160 × 160 nm^2^) of individual IBDV particles are shown (top, right inset). Hexagon diagrams show the amount of RNP (indicated as dots) packed inside the particle for each type of IBDV population (top, left inset). Each dot in the diagrams corresponds to a VP3-dsRNA monomer (with an A or B segment). (**g,h**) Histogram of the slopes of the indentation curves for (**g**) empty and (**h**) full IBDV T = 13 VLP. The k value for each population was calculated by Gaussian fits; for (**g**) k_Emty VLP_ = 0.277 ± 0.088 N/m and (**h**) k_Full VLP_ = 00.320 ± 0.134 N/m. AFM images (160 × 160 nm^2^) of individual IBDV (**a–f)** and T = 13 VLP (**g,h)** are shown (top, right inset).

We evaluated whether differences in dsRNA content influence viral properties such as capsid rigidity, which could mediate viral function and evolution. To analyze these properties, we used AFM nanoindentation^30^ to measure the mechanics of six IBDV populations in physiological conditions. A linear reversible deformation is predicted for indentations of thin homogeneous shells when the indentation is in the order of the shell thickness. In that case, viral capsids are remarkably elastic and their properties are well represented by a continuum elasticity model. Capsid stiffness can hence be derived analytically from indentation experiments. Figure 1a–f shows a gallery of rigidity slopes obtained from indentation experiments with individual intact T = 13 capsids of these IBDV populations. The histogram from E1 T = 13 capsids (empty capsids) summarizes the slope distribution of 130 indentations obtained for 26 IBDV particles (Fig. 1a). Gaussian fitting of these data yielded a spring constant k_E1_ = 0.35 ± 0.12 N/m (mean ± SD).

**Figure 2:**
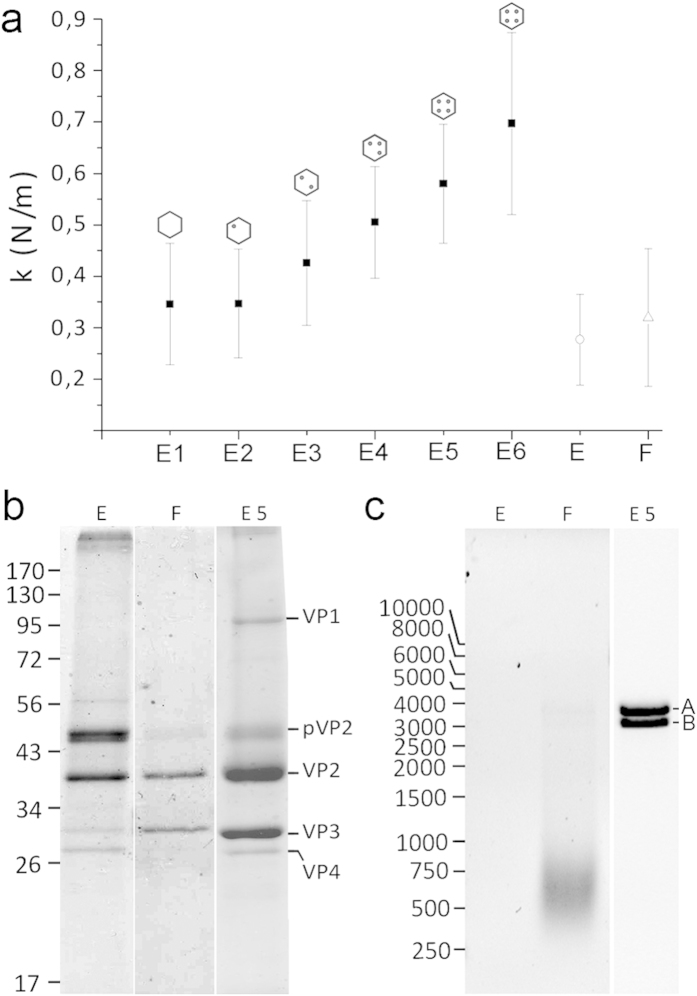
Comparison of the mechanical rigidity of E1-E6 IBDV capsids with empty and full IBDV VLP. (**a**) Mean rigidity values of E1-E6 IBDV capsids, as well as empty (E) and full (F) IBDV-derived T = 13 virus-like particles. (**b**) Coomassie blue-stained SDS-PAGE gels of empty (E) and full (F) VLP, and E5 capsids. Molecular size markers (× 10^−3^ Da) are shown at left; bands corresponding to proteins VP1, pVP2, VP2, VP3 and VP4 are indicated. (**c**) Agarose gel electrophoresis of nucleic acids contained in empty (E) and full (F) VLP, and in E5 particles. Cell ssRNA is seen as a diffuse band (F), and bands of dsRNA A and B segments are indicated. Left, molecular weight markers (bp). Full-length gels are presented in Supplementary Fig. 3.

Equivalent measurements and calculations were used for E2-E6 populations, with 84 to 139 indentations for 18 to 27 particles, respectively (Fig. 1b–f). The results showed that although capsid morphology is indistinguishable in these populations, capsid rigidity increased evenly (k_E2_, 0.35 ± 0.11 N/m; k_E3_, 0.43 ± 0.12 N/m; k_E4_, 0.51 ± 0.11 N/m; k_E5_, 0.58 ± 0.12 N/m; k_E6_, 0.70 ± 0.18 N/m) (Fig. 2a). This increased rigidity correlated with the increasing amounts of packaged genome within each virus population (from empty particles to capsids with four RNP). These data suggest a mechanical signature of the progressive packing of genomic dsRNA.

### Mechanical properties of IBDV viral-like particles (VLP)

During IBDV capsid assembly, the VP3 C-terminal end establishes electrostatic interactions with a pVP2 C-terminal amphipathic α-helix, which is removed proteolytically in most mature virions^16^. Since the genomic dsRNA binds VP3 to build RNP complexes inside virions^11^, the strengthening effect described above might be mediated by the whole complex or by dsRNA or VP3 alone. We compared the mechanical response of IBDV virions with VLP produced by IBDV polyprotein expression in an inducible recombinant vaccinia virus^24^,^31^. In this heterologous system, the IBDV polyprotein is processed correctly and most VLP are assembled with the same shape and size as authentic IBDV particles, as capsids with a T = 13 lattice. We purified VLP by consecutive ultracentrifugations in sucrose and CsCl gradients and obtained two bands with different buoyant densities based on content (Supplementary Fig. 2). The upper band contained empty VLP and the lower band, VLP bearing heterogeneous cellular messengers (ssRNA) that were encapsidated non-specifically. In nanoindentation experiments, these VLP showed rigidities of 0.28 ± 0.09 N/m and 0.32 ± 0.13 N/m for empty and RNA-containing VLP, respectively (Fig. 1g,h). The rigidity values for these VLP were similar to that of the (empty) E1 population (0.35 ± 0.12 N/m) and much lower than those of the other dsRNA-containing IBDV particles (Fig. 2a).

Biochemical analyses showed that empty and full VLP were composed of the same structural proteins as IBDV (except for VP1), with varying amounts of pVP2/VP2 (Fig. 2b, Supplementary Fig. 3). Full VLP contained cellular ssRNA (Fig. 2c, Supplementary Fig. 3). The similarity in rigidity for empty and full VLP suggests that, in the absence of dsRNA (as for E1 IBDV and empty VLP), VP3 is unable to increase the rigidity of the T = 13 capsid. The presence of nonspecific ssRNA within VLP, irrespectively of whether VP3 is free or ssRNA-bound, is irrelevant to capsid mechanical properties. Our results thus suggest a specific role for VP3/dsRNA RNP complexes in the reinforcement of IBDV capsids, although dsRNA alone could also mediate capsid strength.

### Mechanical fatigue in IBDV populations

**Figure 3:**
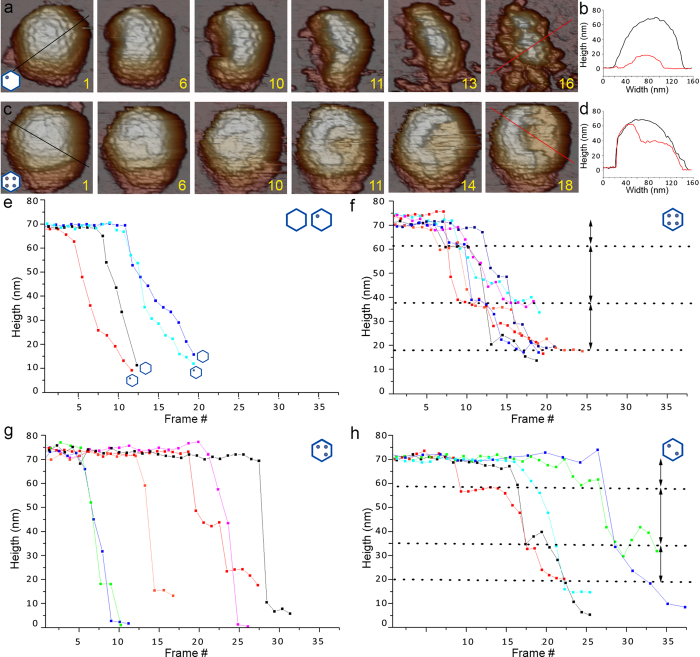
Disruption of E1-E6 IBDV particles by AFM tip mechanical fatigue. Selected frames from capsid dismantling of (**a**) E2 and (**c**) E5 IBDV capsids. Numbers indicate loading cycle number applied to each IBDV particle (movies S1 and S2 show complete image datasets for E2 and E5 particles, respectively). (**b,d**) Black and red profiles (frames 1 and 16 for E2; frames 1 and 18 for E5) show shape evolution of (**c**) E2 and (**d**) E5 capsids during the fatigue experiment. Height changes are indicated by dashed lines. E2 particle height changed from ~70 nm (black line) to ~15 nm (red line); for E5, particle height changed from ~70 nm (black) to ~60 and ~40 nm (red). (e-h) Topographic changes of individual (**e**) E1-E2, (**f**) E5, (**g**) E4 and (**h**) E3 particles (5–7 particles of each IBDV population) during the fatigue experiment. Whereas E1-E2 and E4 (odd number of RNP copies) collapse rapidly after shell damage, E5 and E3 (even RNP copy numbers) show more complex behavior, with a stepwise dismantling sequence (dashed lines).

To characterize the IBDV capsid mechanical reinforcement mediated by VP3/dsRNA RNP, we studied capsid response to mechanical fatigue that consisted of repeated loading forces below the breaking force (~150 pN), which allow controlled peeling of the virus shell and direct access to the internal RNP core^32^. Continuous imaging of a single virus in jumping mode^33^ produces cyclic loading at low force, induces stepwise disruption of individual IBDV particles, and permits simultaneous real-time monitoring of disassembly. The IBDV capsid is ~10 nm thick and has an internal radius of ~26 nm. The sequence of dismantling events was characteristic for each IBDV population, which began to release fragments whose thickness is similar to that of the capsid shell, followed by core exposure (Fig. 3 and Supplementary Fig. 4).

Fatigue behavior was similar for E1 and E2 particles. In a representative example, we scanned an E2 particle (one RNP) 16 times (~64 min) (Fig. 3a). Frame 1 shows the intact particle ~68 nm high virions (Fig. 3b, black line). Frames 6–11 showed progressive capsid dismantling that originated in a small fracture, leading to particle crumbling (frame 13). After frame 14, the particle collapsed to ~20 nm high debris (frame 16; Fig. 3b, red line). The results suggest that the induced fatigue first removed some capsomers, which weakened the viral shell and led to its complete disruption (Supplementary Movie 1).

The behavior of particles containing four dsRNA molecules differed from these E2 particles. Mechanical fatigue of E5 (E5 and E6 constitute ~64% of the total virus population and will be referred below as E5, as both have four RNP) showed defined stepwise dismantling over 64 min (Fig. 3c). In the same experimental fatigue protocol, E5 disruption began with capsomer dissociation as suggested for E2 (frames 6–10). Real-time monitoring showed no subsequent particle collapse, and structural integrity was maintained. The upper part of the capsid that probably corresponds to the shell then suddenly separated from the virus (frames 11–14) in a process in which a number of capsomers collectively peeled off the capsid. Height was maintained at ~35 nm (frame 18; Fig. 3d) with exposure of the internal core, which was stable within the remaining shell. This behavior suggests that the RNP stabilizes capsid structure and prevents particle destruction, even in conditions in which a large part of the shell is absent (Supplementary Movie 2).

Systematic study of the IBDV populations thus showed specific fatigue responses for each population (Supplementary Fig. 4a–d). Based on particle height during mechanical fatigue analysis, E1 and E2 particles showed “capsid cracking”, a breakage pattern characterized by rapid destruction following capsid fracture (Fig. 3e). Although fracture starting time varied from particle to particle, the linear breakage profile was similar. The topographical destruction profile was very different for E5 particles, in which mechanical fatigue induced stepwise dismantling of particles (Fig. 3f). This process is simplified here to three steps in E5 particle dismantling (dotted lines), with removal of the outer shell (~10 nm) and two consecutive ~20 nm structures, until the remaining shell attached to the substrate was reached.

Fatigue experiments for E3 and E4 (2 and 3 RNP, respectively) yielded distinct fatigue profiles (Supplementary Fig. 4b,c). Whereas E4 particles had a continuous linear destruction profile (Fig. 3g) similar to those of E1 and E2 (Fig. 3e), the E3 particles behaved like E5 particles, with a three-step topographical disruption profile (Fig. 3h). Breaking time for many E4 particles was delayed relative to E1 and E2 particles, which indicated E4 capsid strengthening mediated by RNP complexes.

### IBDV RNP dimers and higher order complexes

**Figure 4:**
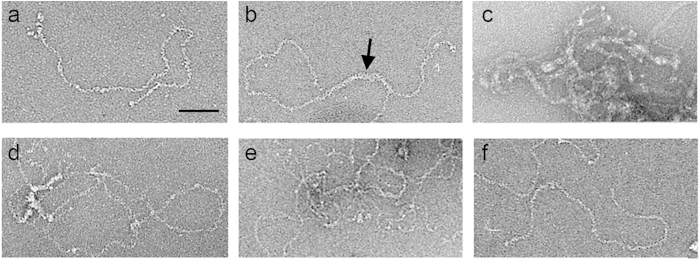
Reversible assembly of IBDV RNP dimers and higher order complexes. Electron micrographs of negatively stained (**a**) RNP in TE buffer (5 mM Tris-HCl pH 8.0, 5 mM EDTA), (**b**) RNP incubated with 2 mM CaCl_2_ (2 h), (**c**) RNP incubated with 5 mM CaCl_2_ (2 h), (**d**) RNP as in (**c**) incubated with 5 mM EDTA (2 h), (**e**) RNP as in (**c**) incubated with 10 mM EDTA (2 h), and (**f**) RNP as in (**c**) incubated with 20 mM EDTA (2 h).

The existence of two similar steps (with the same height) in the E3 and E5 topological profiles, which are populations presenting an even RNP copy number, is consistent with two sequential intermediates during dismantling. The absence of these two steps in E2 and E4 profiles (both with an odd RNP copy number) raises the question as to how packaged dsRNA/VP3 complexes are organized to influence virus stability so differently. We tested whether RNP self-interact to form larger complexes, compatible with the AFM induced fatigue results. Electron microscopy of negative stained, purified RNP showed a “rope ladder” appearance, in which steps would correspond to VP3 wrapping the flexible dsRNA filaments homogeneously. When purified RNP (Fig. 4a) was incubated with 2 mM divalent cations (Ca^2+^ or Mg^2+^), the single RNP filaments (~30–40 nm diameter) interacted side-by-side to yield partially dimeric assemblages (~70–90 nm diameter) (Fig. 4b, arrow). At 5 mM, these cations caused a tangled aggregation of RNP filaments (Fig. 4c). This process was reversible, as incubation of 5 mM Ca^2+^ or Mg^2+^-induced RNP aggregates with increasing concentrations of a chelating agent such as EDTA led to progressive resolution of aggregates into single-filament RNP (Fig. 4d–f). Single RNP filaments (Fig. 4a,f) had mean lengths of 0.88 and 0.77 μm. These values resemble an A-type duplex (pitch 2.81–3 Å), since IBDV dsRNA segments A and B measure 0.9–0.96 and 0.79–0.84 μm, as VP3 bound to dsRNA does not condense the filament^11^. These results are consistent with reversible formation of higher order RNP complexes, probably dimers, which might explain the stepwise disruption profile of mature virions (E5) and could have a role in capsid maturation and disassembly.

## Discussion

Viruses use diverse genetic strategies and life cycles to parasitize host cellular machinery. The virus genetic program produces multiple copies of infectious particles, although the ratio of defective particles during the replication cycle varies from 1 in 10 to 1 in 10,000 (or more) [see^34^,^35^]. For IBDV, E4-E6 populations have more than one complete genome and make up ~80% of total particles. E5-E6 constitute ~64% and have the greatest viral fitness, with a particle/plaque-forming unit ratio of approximately six^8^. The availability of IBDV populations with different genome contents (some empty and/or defective) allowed us to determine whether mechanical properties such as elastic strength and resilience are linked to infectivity and/or provide additional selective advantages. Our analysis indicates that increased genome content in the virion reinforces capsid rigidity; the higher tolerance to external stress also contributes to greater infectivity.

Viral nucleic acids have a structural role in the assembly and stabilization of many viruses. Capsid assembly can proceed spontaneously by self-assembly of capsid proteins, or with the assistance of other components such as scaffolding proteins or nucleic acids in a condensation process^36^. For many viruses (especially ssRNA and some dsDNA viruses such as simian virus 40), the capsid self-assembles around the viral genome. Electrostatic interactions between positive charges on capsid proteins and negative charges on RNA provide an important thermodynamic driving force for this process^37^. Some ssRNA and dsRNA viruses have genome regions that interact with capsid proteins at symmetrically equivalent positions^38^. The average structure of these ordered regions has been visualized at high resolution as a duplex RNA. Optimal genome length is determined by a complex interplay between charge, capsid size, excluded-volume and RNA structure^39^,^40^. Secondary structure predictions suggest that viral RNA have a compact tertiary structure, which could promote assembly around the viral RNA^41^. In addition, unfavorable nucleic acid features can direct assembly toward aberrant capsid morphologies^42^.

AFM indentation analyses of dsDNA viruses (such as phage ϕ29) showed that the tightly packaged dsDNA (~45 bp/100 nm^3^, with high internal pressure of ~40–60 atm) increases particle rigidity, like the compressed air in a soccer ball^43^. In minute virus of mice (MVM), the ssDNA interacts specifically with residues that face the internal capsid surface and has a buttressing effect^7^,^44^. Triatoma virus, a ssRNA virus with packing density higher than that of ϕ29 and λ phages, shows electrostatic interactions between RNA and positively-charged patches on the inner capsid wall^45^. In naturally occurring IBDV E5 particles (dsRNA density ~20 bp/100 nm^3^), greater mechanical rigidity is associated with an increase in the packaged dsRNA/VP3 RNP complexes. RNP stabilize the capsid and help to maintain the shell structural integrity, even if some capsomers are lost.

**Figure 5:**
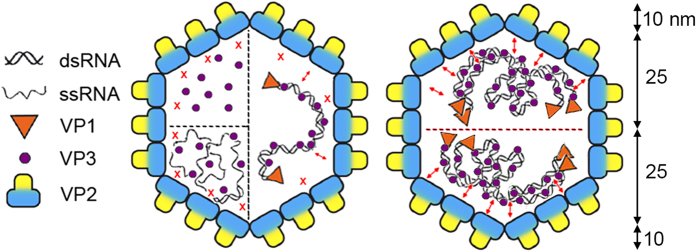
RNP dimers are the basic stabilization units of the IBDV capsid. Scheme of the IBDV capsid showing structural components involved in stabilizing interactions. When VP3 is found alone inside the capsid (as in empty VLP, E1 and E2) or in the presence of ssRNA (as in full VLP), contacts with VP2 (red X) are weak or non-existent. When dsRNA is present, these contacts are more intense (small double arrowheads), presumably because dsRNA-bound VP3 acquire the correct conformation to establish a stronger interaction with VP2 (left). Mature IBDV virions, represented by E5-E6 capsids, are polyploid and contain four packaged dsRNA segments organized as RNP. In this case, RNP are organized internally as dimers; in this oligomeric state, VP3 acquires a conformation with which it establishes strong interactions with the inner surface of the viral shell. This structural organization of the genome explains the stepwise disruption profile during mechanical fatigue (shell thickness is ~10 nm based on three-dimensional cryo-EM analysis).

RNase digestion assays of purified RNP suggested that VP3 shields dsRNA homogeneously^11^, and we assume that dsRNA-bound VP3 probably mediates the mechanical resilience of the IBDV capsid, although dsRNA might also be involved. VP3, alone or in the presence of nonspecific cellular ssRNA (free or ssRNA-bound), does not increase capsid rigidity. These results imply that VP3 acquires a conformation competent for interaction with VP2 only by binding to dsRNA (Fig. 5). The VP2-VP3 interaction differs from pVP2-VP3 interactions during capsid assembly (see below). Three-dimensional reconstructions of virions showed only capsid densities corresponding to VP2, with no trace of residual VP3-related densities^9^,^46^, which suggests that the VP2-VP3 interactions detected in AFM analysis are random and/or stable but irregular. Study of VP3 mutants that partially affect capsid stability without altering other activities such as dsRNA binding could identify the VP3 segments involved in post-assembly capsid stabilization.

VP3 is a moonlighting protein with several functions in the viral life cycle. VP3 in IBDV virions has a relatively constant copy number of ~450, independently of the number of packaged dsRNA segments^8^. VP3 incorporation thus appears to be determined mainly by its role as a scaffolding protein during assembly of pVP2 immature capsid proteins into a provirion particle^20^. Analysis of E1-E6 RNP content by native agarose gel electrophoresis indicates that VP3 is bound to dsRNA at a constant ratio, as defined bands are detected that correspond to RNP-A and RNP-B^11^. This stoichiometry for VP3 implies indirectly that many VP3 molecules remain unbound to dsRNA molecules as “soluble proteins” in the capsid interior; this is particularly the case of E1-E3 T = 13 capsids that have 0, 1 or 2 dsRNA molecules, respectively. The last five residues of the acidic VP3 C-terminal region interact with the transiently bound ^443^GFKDIIRAIR^452^ amphipathic α-helix of pVP2^20^. Once virions are nearly or recently assembled as provirions, VP3 is probably freed from its scaffold function when the pVP2 C-terminal region is proteolyzed by VP4, by a cell protease^16^ or by VP2 itself during virus maturation^17^. VP3 then switches to RNA-binding protein and VP1 activator functions and, in the mature virion, binds to dsRNA^11^.

Here we identified an additional VP3-mediated capsid-stabilizing function, presumably involving different VP3 regions than those used during capsid assembly. VP3 thus has a simultaneous double scaffolding role, by interacting with the mature form of the capsid protein VP2 and by stabilizing both segments of the genomic dsRNA. The switching mechanisms among the various VP3 functions are poorly understood. These activities might be coordinated by conformational changes in partner-induced processes (pVP2, VP2, VP1, dsRNA, or their combinations), by variations in oligomeric state or protein modification, by transient folded/unfolded states, local ligand concentration at the viroplasm, or a combination of these.

We used mechanical fatigue to obtain information on the virus disassembly pathway that guarantees successful infection^32^. IBDV populations were subjected to a sequence of constant loading forces to induce gradual modification in the virion molecular structure. Our analysis showed a major IBDV feature, the existence of two distinct disruption patterns, continuous or stepwise. E5 particles package four RNP and are dismantled in approximately three steps; after removal of a number of capsomers, a shell fragment whose thickness probably corresponds to that of the capsid is lost and the RNP core is exposed. In this situation, the top half of the core is lost first, followed by the bottom half. This (four)-RNP core organization in two halves would be consistent with RNP assembly as two dimers in the capsid interior. We induced progressive RNP interactions *in vitro* by varying the Ca^2+^ or Mg^2+^ concentration. VP3 atomic structure shows that its central region (residues 92–220), which is folded into two α-helical domains connected by a long hinge, is organized as a dimer^18^. The electrostatic potential of VP3 dimer interacting surfaces shows highly negatively charged areas that would be neutralized by divalent cations and/or high salt concentrations.

E3 and E5 respond similarly to mechanical fatigue (although E3 are more variable), and both have an even number of RNP complexes that could assemble as one or two dimers, respectively. E4, which packages three RNP, is destroyed by mechanical stress following a linear destruction profile; this resemblance to E1-E2 particles suggests that three RNP molecules do not constitute an optimal configuration for RNP dimer formation. These data highlight the difference between shell structure rigidity and ductility. Although E4 capsids showed greater rigidity than E3, they were less resistant to mechanical fatigue, as the RNP stabilizing effect was reduced. We speculate that this scaffolding role is mediated by VP3 dimers bound to dsRNA filaments.

In conclusion, our AFM studies of IBDV, combined with previous structural analyses from three-dimensional cryo-EM and X-ray crystallography, show a direct correlation between IBDV capsid mechanical rigidity and the number of viral RNPs incorporated into the virion capsid. These characteristics might be mediated by higher order RNP oligomers and have important implications in the virus life cycle. The IBDV system provides a simple model for study of the molecular basis by which a protein moonlights between different functions and performs several activities simultaneously.

## Methods

### Cells and viruses

IBDV infections were carried out with the Soroa isolate^24^ adapted for growth in chicken embryonic fibroblast primary cultures. It was then adapted for growth in QM7 quail muscle cells. Recombinant vaccinia virus (rVV) VT7/POLY has been described^24^. QM7 cells were cultured in Dulbecco’s modified Eagle’s medium with 10% fetal calf serum.

### Virion purification

QM7 cells were infected with IBDV Soroa strain at a multiplicity of infection (MOI) of 1–2 plaque-forming units (pfu) per cell. IBDV particles from cell medium were harvested 72 h postinfection (hpi), precipitated with 3.5% polyethylene glycol 8000 and 0.5 M NaCl, and the resulting pellet was resuspended in PES buffer [25 mM piperazine-N,N_-bis(2-ethanesulfonic acid) pH 6.2, 150 mM NaCl, 20 mM CaCl_2_]. Particles were pelleted through a 25% (w/w) sucrose cushion (170,000 × g, 150 min, 4 °C). The initial density of the solution was adjusted to 1.33 g/ml by adding CsCl, followed by CsCl equilibrium gradient centrifugation (130,000 × g, 14 h, 4 °C). Six virus-containing bands were visible (designated E1 to E6, from top to bottom), and were collected separately by side puncturing. Fractions were dialyzed against PES buffer and stored at 4 °C for a maximum of 2–3 weeks.

### Purification of IBDV polyprotein-derived VLP

QM7 cells were infected with the rVV VT7/POLY at a MOI of 1–5 pfu/cell. Cells were harvested at 60 hpi, resuspended in PES buffer with 1 mM PMSF, and subjected to three of freeze-thaw and sonication cycles. The lysate was clarified by centrifugation (1000 × g, 10 min), and the supernatant processed on a 25% sucrose cushion (170,000 × g, 150 min, 4 °C). The pellet was resuspended in PES buffer, centrifuged in a microfuge (16,000 × g, 1 min), and the supernatant processed on a linear 25–50% sucrose gradient (200,000 × g, 45 min, 4 °C). The particulate material containing polyprotein-derived structures was collected in 12 fractions and concentrated 20-fold by ultracentrifugation (240,000 × g, 120 min, 4 °C). VLP-enriched fractions were pooled, the initial density of the solution was adjusted to 1.31 g/ml by adding CsCl and then centrifuged on a CsCl equilibrium gradient (130,000 × g, 14 h, 4 °C).

### Purification of RNP and dsRNA from IBDV virions, and purification of cell RNA from full IBDV VLP

RNP complexes were purified from disrupted E5 viral particles obtained by dialysis against a low ionic strength buffer (5 mM Tris-HCl pH 8.0, 5 mM EDTA)(72 h, 20 °C). This extract (~200 μl) was centrifuged twice on a glycerol step gradient [0.5 ml 70% (w/v) glycerol, 0.75 ml 50% glycerol, 0.375 ml 40% glycerol, 1.2 ml 33% glycerol] (150,000 × g, 3 h, 20 °C). Fractions 5–7 (of 10 fractions, 300 μl each) were enriched in RNP. When the glycerol gradient buffer contained 10 mM CaCl_2_, RNP-enriched fractions were located in the bottom fractions.

E5 particles were incubated with 1% SDS (3 min, 98 °C; 40 s, 4 °C) and treated with proteinase K (2 mg/ml, 2 h, 37 °C) for dsRNA analysis. dsRNA was extracted with TriZol (Invitrogen) and purified using silica-based mini-spin columns (Qiagen). Cell RNA from full IBDV VLP was purified as for IBDV dsRNA.

### SDS-PAGE

Concentrated gradient fractions (2–5 μl) were added to Laemmli simple buffer to a 1× final concentration (62.5 mM Tris–HCl, 2% SDS, 5% glycerol, 0.012% bromophenol blue, 2 mM dithiothreitol, pH 6.8), heated (3 min, 100 °C), and resolved in 11% polyacrylamide gels.

### Agarose gel electrophoresis

Purified nucleic acids were incubated with 6× blue loading dye (0.25% bromophenol blue, 30% glycerol). Samples were loaded onto 0.7% agarose gels in 90 mM Tris–HCl pH 8.0, 90 mM boric acid and 20 mM EDTA. Electrophoresis was carried out at 20 °C in the same buffer until the dye reached the gel bottom. Samples were visualized with ethidium bromide.

### Electron microscopy

Samples (2–5 μl) were applied to glow-discharged carbon-coated grids (2 min) and negatively stained with 2% aqueous uranyl acetate. Micrographs were recorded with a JEOL 1200 EXII electron microscope operating at 100 kV at a nominal magnification of ×25,000 (for RNP) and ×40,000 (for viruses and VLP).

### Nanoindentation experiments

Surface-attached capsids were imaged in physiological conditions using jumping mode, which allowed accurate control and limitation of maximal tip-sample forces. We used 0.05 N/m force constant Olympus silicon nitride cantilevers. Data were analyzed with WSxM software 47. Capsid position was determined in low resolution images (128 × 128 pixels) at maximal scanning force (<100 pN). The applied force was first calibrated by single Force vs. Z piezo displacement curves (FZ) on a highly-orientated pyrolytic graphite surface (HOPG) next to the capsid. After individual details of capsids were resolved, indentation measurements were performed to quantitate their elastic response. Another profile scan of the capsid was obtained and its height determined. If images acquired immediately after indentation showed no evidence of damage, additional FZ curves were obtained on the same capsid. A series of three to five successive FZ curves was obtained, and measurements performed on 21 to 28 particles (89 to 131 indentations) for each population. No force was observed until the tip contacted the capsid, after which force then rose linearly as tip displacement in z direction decreased to ~20 nm, which corresponds to a compression/deformation of approximately twice the thickness of the viral shell (9 nm). In the region in which force rises linearly, the nanoindentation process was almost completely reversible. Inversion of the direction of tip motion when the cantilever dropped to its initial position resulted in a force that returned along the same straight line.

Small virus and cantilever spring constants can be modeled by two springs in series. The spring constant for the cantilever was determined by calibration on the substrate. From the slope of the FZ curves, the spring constant of the capsids (k_shell_) could be calculated as k_shell_ = k_c_k_eff_(k_c_ – k_eff_)^−1^, where k_eff_ is the effective spring constant due to cantilever bending and shell deformation. As a control, we confirmed that spring constants in successive indentations of a single E1 capsid differed by <10%. The distribution variability for each capsid array is probably due to differences in measurement geometry.

For mechanical fatigue analysis of IBDV populations, particles adsorbed on the HOPG substrate were imaged in buffer conditions by AFM scanning in jumping mode, which required consecutive loading cycles at a maximum constant force of 150 pN.

## Additional Information

**How to cite this article**: Mertens, J. *et al.* A protein with simultaneous capsid scaffolding and dsRNA-binding activities enhances the birnavirus capsid mechanical stability. *Sci. Rep.* **5**, 13486; doi: 10.1038/srep13486 (2015).

## Supplementary Material

Supplementary Movie 1

Supplementary Movie 2

Supplementary Figures

## References

[b1] Harrison S. C. in Fields virology Vol. 1 (eds Knipe D. M. *et al.* ) 59–98 (Lippincott Williams & Wilkins, 2007).

[b2] Baker T. S., Olson N. H. & Fuller S. D. Adding the Third Dimension to Virus Life Cycles: Three-Dimensional Reconstruction of Icosahedral Viruses from Cryo-Electron Micrographs. Microbiol. Mol. Biol. Rev. 63, 862–922 (1999). 10585969 10.1128/mmbr.63.4.862-922.1999 PMC98980

[b3] Trask S. D., Ogden K. M. & Patton J. T. Interactions among capsid proteins orchestrate rotavirus particle functions. Curr. Opin. Virol. 2, 373–379 (2012). 22595300 10.1016/j.coviro.2012.04.005 PMC3422376

[b4] Johnson J. E. Functional implications of protein-protein interactions in icosahedral viruses. Proc. Natl. Acad. Sci. UA 93, 27–33 (1996). 10.1073/pnas.93.1.27 PMC40172 8552620

[b5] Hernando-Perez M., Lambert S., Nakatani-Webster E., Catalano C. E. & de Pablo P. J. Cementing proteins provide extra mechanical stabilization to viral cages. Nat. Commun. 5, 4520 (2014). 25072871 10.1038/ncomms5520

[b6] Roos W. H., Bruinsma R. & Wuite G. J. L. Physical virology. Nat. Physics 6, 733–743 (2010).

[b7] Carrasco C., Castellanos M., de Pablo P. J. & Mateu M. G. Manipulation of the mechanical properties of a virus by protein engineering. Proc. Natl. Acad. Sci. USA 105, 4150–4155 (2008). 18334651 10.1073/pnas.0708017105 PMC2393779

[b8] Luque D. *et al.* Infectious bursal disease virus is an icosahedral polyploid dsRNA virus. Proc. Natl. Acad. Sci. USA 106, 2148–2152 (2009). 19164552 10.1073/pnas.0808498106 PMC2650107

[b9] Coulibaly F. *et al.* The birnavirus crystal structure reveals structural relationships among icosahedral viruses. Cell 120, 761–772 (2005). 15797378 10.1016/j.cell.2005.01.009

[b10] Saugar I. *et al.* Structural polymorphism of the major capsid protein of a double-stranded RNA virus: an amphipathic alpha helix as a molecular switch. Structure 13, 1007–1017 (2005). 16004873 10.1016/j.str.2005.04.012

[b11] Luque D. *et al.* Infectious Bursal disease virus: ribonucleoprotein complexes of a double-stranded RNA virus. J. Mol. Biol. 386, 891–901 (2009). 19063900 10.1016/j.jmb.2008.11.029 PMC7173181

[b12] Hjalmarsson A., Carlemalm E. & Everitt E. Infectious pancreatic necrosis virus: identification of a VP3-containing ribonucleoprotein core structure and evidence for O-linked glycosylation of the capsid protein VP2. J. Virol. 73, 3484–3490 (1999). 10074207 10.1128/jvi.73.4.3484-3490.1999 PMC104117

[b13] Feldman A. R., Lee J., Delmas B. & Paetzel M. Crystal structure of a novel viral protease with a serine/lysine catalytic dyad mechanism. J. Mol. Biol. 358, 1378–1389 (2006). 16584747 10.1016/j.jmb.2006.02.045

[b14] Birghan C., Mundt E. & Gorbalenya A. E. A non-canonical lon proteinase lacking the ATPase domain employs the Ser-Lys catalytic dyad to exercise broad control over the life cycle of a double-stranded RNA virus. EMBO J. 19, 114–123 (2000). 10619850 10.1093/emboj/19.1.114 PMC1171783

[b15] Sánchez A. B. & Rodríguez J. F. Proteolytic processing in infectious bursal disease virus: identification of the polyprotein cleavage sites by site-directed mutagenesis. Virology 262, 190–199 (1999). 10489352 10.1006/viro.1999.9910

[b16] Irigoyen N., Castón J. R. & Rodríguez J. F. Host proteolytic activity is necessary for infectious bursal disease virus capsid protein assembly. J. Biol. Chem. 287, 24473–24482 (2012). 22619177 10.1074/jbc.M112.356113 PMC3397872

[b17] Irigoyen N. *et al.* Autoproteolytic activity derived from the infectious bursal disease virus capsid protein. J. Biol. Chem. 284, 8064- 8072 (2009). 19144647 10.1074/jbc.M808942200 PMC2658100

[b18] Casañas A. *et al.* Structural insights into the multifunctional protein VP3 of birnaviruses. Structure 16, 29–37 (2008). 18184581 10.1016/j.str.2007.10.023

[b19] Maraver A. *et al.* The oligomerization domain of VP3, the scaffolding protein of infectious bursal disease virus, plays a critical role in capsid assembly. J. Virol. 77, 6438–6449 (2003). 12743301 10.1128/JVI.77.11.6438-6449.2003 PMC155005

[b20] Saugar I. *et al.* Electrostatic interactions between capsid and scaffolding proteins mediate the structural polymorphism of a double-stranded RNA virus. J. Biol. Chem. 285, 3643–3650 (2010). 19933276 10.1074/jbc.M109.075994 PMC2823505

[b21] Oña A. *et al.* The C-terminal domain of the pVP2 precursor is essential for the interaction between VP2 and VP3, the capsid polypeptides of infectious bursal disease virus. Virology 322, 135–142 (2004). 15063123 10.1016/j.virol.2004.01.025

[b22] Maraver A., Clemente R., Rodríguez J. F. & Lombardo E. Identification and molecular characterization of the RNA polymerase-binding motif of infectious bursal disease virus inner capsid protein VP3. J. Virol. 77, 2459–2468 (2003). 12551984 10.1128/JVI.77.4.2459-2468.2003 PMC141113

[b23] Garriga D. *et al.* Activation mechanism of a noncanonical RNA-dependent RNA polymerase. Proc. Natl. Acad. Sci. USA 104, 20540–20545 (2007). 18077388 10.1073/pnas.0704447104 PMC2154467

[b24] Lombardo E. *et al.* VP1, the putative RNA-dependent RNA polymerase of infectious bursal disease virus, forms complexes with the capsid protein VP3, leading to efficient encapsidation into virus-like particles. J. Virol. 73, 6973–6983 (1999). 10400796 10.1128/jvi.73.8.6973-6983.1999 PMC112783

[b25] van Cleef K. W. *et al.* Mosquito and Drosophila entomobirnaviruses suppress dsRNA- and siRNA-induced RNAi. Nucleic Acids Res. 42, 8732–8744 (2014). 24939903 10.1093/nar/gku528 PMC4117760

[b26] Tompa P., Szasz C. & Buday L. Structural disorder throws new light on moonlighting. Trends Biochem. Sci. 30, 484–489 (2005). 16054818 10.1016/j.tibs.2005.07.008

[b27] Mani M. *et al.* MoonProt: a database for proteins that are known to moonlight. Nucleic Acids Res. 43, D277–282 (2015). 25324305 10.1093/nar/gku954 PMC4384022

[b28] Communie G., Ruigrok R. W., Jensen M. R. & Blackledge M. Intrinsically disordered proteins implicated in paramyxoviral replication machinery. Curr. Opin. Virol. 5, 72–81 (2014). 24631901 10.1016/j.coviro.2014.02.001

[b29] Bellamy A. R. & Joklik W. K. Studies on the A-rich RNA of reovirus. Proc. Natl. Acad. Sci. USA 58, 1389–1395 (1967). 5237873 10.1073/pnas.58.4.1389 PMC223936

[b30] Hernando-Perez M. *et al.* The interplay between mechanics and stability of viral cages. Nanoscale 6, 2702–2709 (2014). 24452242 10.1039/c3nr05763a

[b31] Castón J. R., Rodríguez J. F. & Carrascosa J. L. in Segmented double-stranded RNA viruses. Structure and molecular biology (ed Patton J. T. ) 133–144 (Caister Academic Press, 2008).

[b32] Ortega-Esteban A. *et al.* Monitoring dynamics of human adenovirus disassembly induced by mechanical fatigue. Sci. Rep. 3, 1434 (2013). 23486377 10.1038/srep01434 PMC3595926

[b33] Ortega-Esteban A. *et al.* Minimizing tip-sample forces in jumping mode atomic force microscopy in liquid. Ultramicroscopy 114, 56–61 (2012). 22356789 10.1016/j.ultramic.2012.01.007

[b34] van der Schaar H. M. *et al.* Characterization of the early events in dengue virus cell entry by biochemical assays and single-virus tracking. J. Virol. 81, 12019–12028 (2007). 17728239 10.1128/JVI.00300-07 PMC2168764

[b35] Carpenter J. E., Henderson E. P. & Grose C. Enumeration of an extremely high particle-to-PFU ratio for Varicella-zoster virus. J. Virol. 83, 6917–6921 (2009). 19369328 10.1128/JVI.00081-09 PMC2698559

[b36] Mateu M. G. Assembly, stability and dynamics of virus capsids. Arch. Biochem. Biophys. 531, 65–79 (2013). 23142681 10.1016/j.abb.2012.10.015

[b37] Perlmutter J. D. & Hagan M. F. Mechanisms of virus assembly. Annu. Rev. Phys. Chem. 66, 217–239 (2015). 25532951 10.1146/annurev-physchem-040214-121637 PMC4382372

[b38] Schneemann A. The structural and functional role of RNA in icosahedral virus assembly. Annu. Rev. Microbiol. 60, 51–67 (2006). 16704342 10.1146/annurev.micro.60.080805.142304

[b39] Perlmutter J. D., Qiao C. & Hagan M. F. Viral genome structures are optimal for capsid assembly. eLife 2, e00632 (2013). 23795290 10.7554/eLife.00632 PMC3683802

[b40] Zandi R. & van der Schoot P. Size regulation of ss-RNA viruses. Biophys. J. 96, 9–20 (2009). 18931258 10.1529/biophysj.108.137489 PMC2710049

[b41] Yoffe A. M. *et al.* Predicting the sizes of large RNA molecules. Proc. Natl. Acad. Sci. USA 105, 16153–16158 (2008). 18845685 10.1073/pnas.0808089105 PMC2570976

[b42] Zlotnick A., Porterfield J. Z. & Wang J. C. To build a virus on a nucleic acid substrate. Biophys. J. 104, 1595–1604 (2013). 23561536 10.1016/j.bpj.2013.02.005 PMC3617413

[b43] Hernando-Perez M. *et al.* Direct measurement of phage phi29 stiffness provides evidence of internal pressure. Small 8, 2366–2370 (2012). 22648860 10.1002/smll.201200664

[b44] Carrasco C. *et al.* DNA-mediated anisotropic mechanical reinforcement of a virus. Proc. Natl. Acad. Sci. USA 103, 13706–13711 (2006). 16945903 10.1073/pnas.0601881103 PMC1564217

[b45] Snijder J. *et al.* Probing the biophysical interplay between a viral genome and its capsid. Nat. Chem. 5, 502–509 (2013). 23695632 10.1038/nchem.1627

[b46] Luque D. *et al.* Infectious bursal disease virus capsid assembly and maturation by structural rearrangements of a transient molecular switch. J. Virol. 81, 6869–6878 (2007). 17442720 10.1128/JVI.00077-07 PMC1933288

[b47] Horcas I. *et al.* WSXM: a software for scanning probe microscopy and a tool for nanotechnology. Rev. Sci. Instrum. 78, 013705 (2007). 17503926 10.1063/1.2432410

