# Supplementary material for: A protein with simultaneous capsid scaffolding and dsRNA-binding activities enhances the birnavirus capsid mechanical stability
Source: Sci Rep. 2015 Sep 4;5:13486. doi: 10.1038/srep13486 (PMC4559658; doi:10.1038/srep13486)
Supplement: Supplementary Figures [file srep13486-s3.pdf]

Supplementary Figures

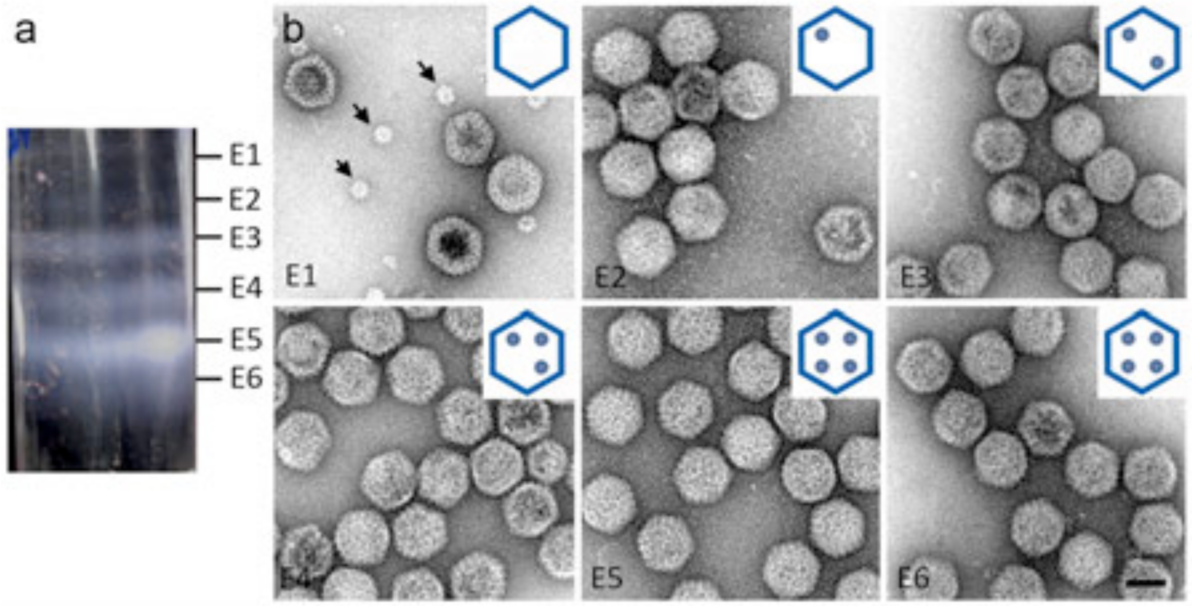

Supplementary Figure 1. Purification of IBDV natural populations. (a) A CsCl linear gradient for IBDV purification illuminated from the bottom after centrifugation to equilibrium containing at least six IBDV fractions (denoted E1 to E6, from top to bottom). E1-E6 bands represent 3, 5, 12, 16, 48 and 16% of the total virion particles. E6 have a slightly higher buoyant density, probably due to the additional presence of unspecific viral RNA fragments or, even, cellular RNA as described in other viruses; E4-E5 populations that contain more than 1 complete genome account for ~80% of total particles. (b) Electron microscopy of purified E1-E6 populations negatively stained with 2% uranyl acetate. T=1 particles (arrows) are indicated for E1 population. The diagrams show the amount of RNP (indicated as dots; see Fig. 1) packed within the particle for each type of IBDV population (top, right inset). Bar, 50 nm.

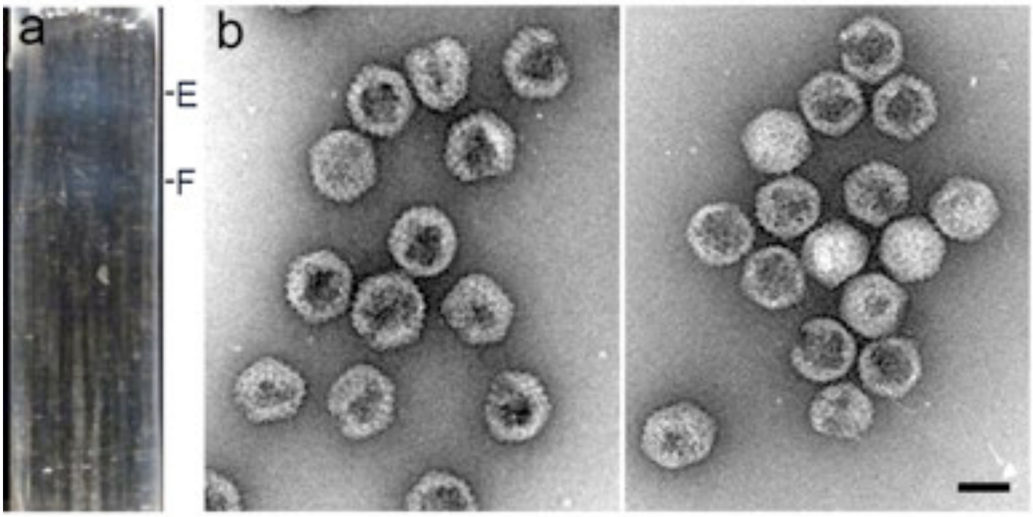

Supplementary Figure 2. Purification of IBDV VLP from rVV VT7/POLY. (a) A CsCl linear gradient for IBDV VLP purification illuminated from the bottom after centrifugation to equilibrium containing at least two VLP fractions (denoted E for empty VLP and F for full VLP , from top to bottom). (b) Electron microscopy of purified empty (left) and full (right) VLP negatively stained with 2% uranyl acetate. Bar, 50 nm.

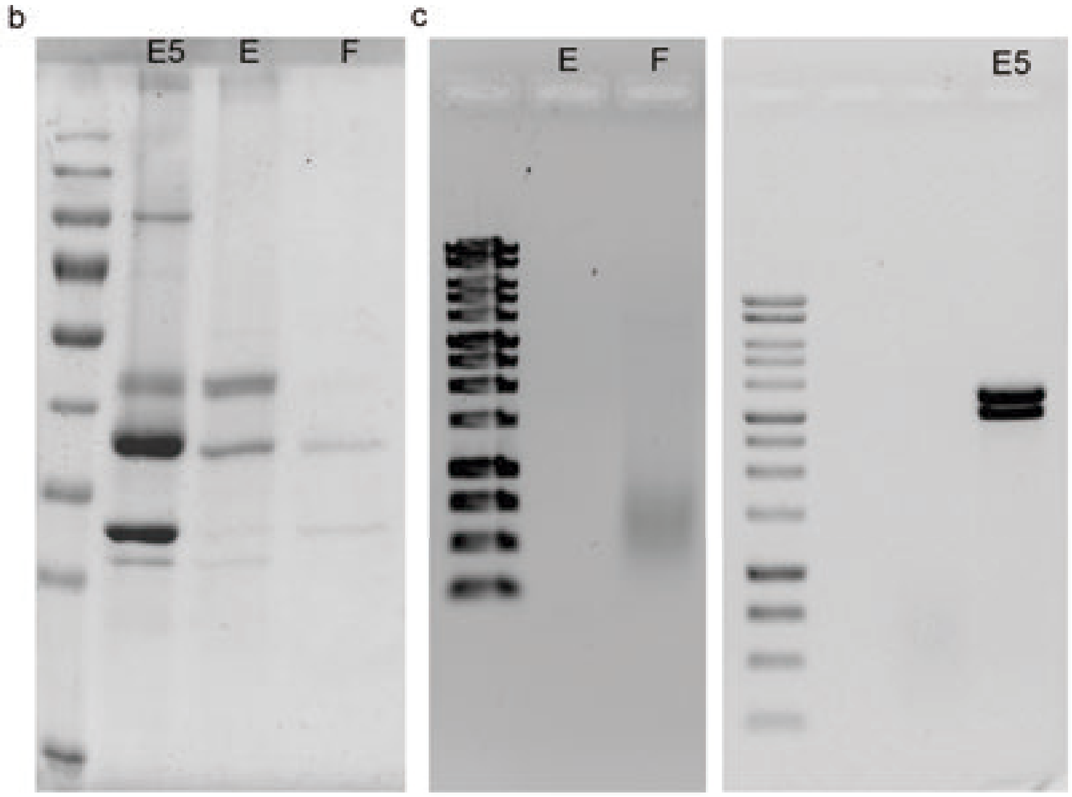

Supplementary Figure 3. (b) Coomassie blue-stained SDS-PAGE gel of empty (E) and full (F) VLP, and E5 capsids. Molecular size markers (x 10^{-3} Da) are shown at left (as indicated in Fig. 2b). (c) Agarose gel electrophoresis of nucleic acids contained in empty (E) and full (F) VLP, and in E5 particles. Left, molecular weight markers (as indicated in Fig. 2c).

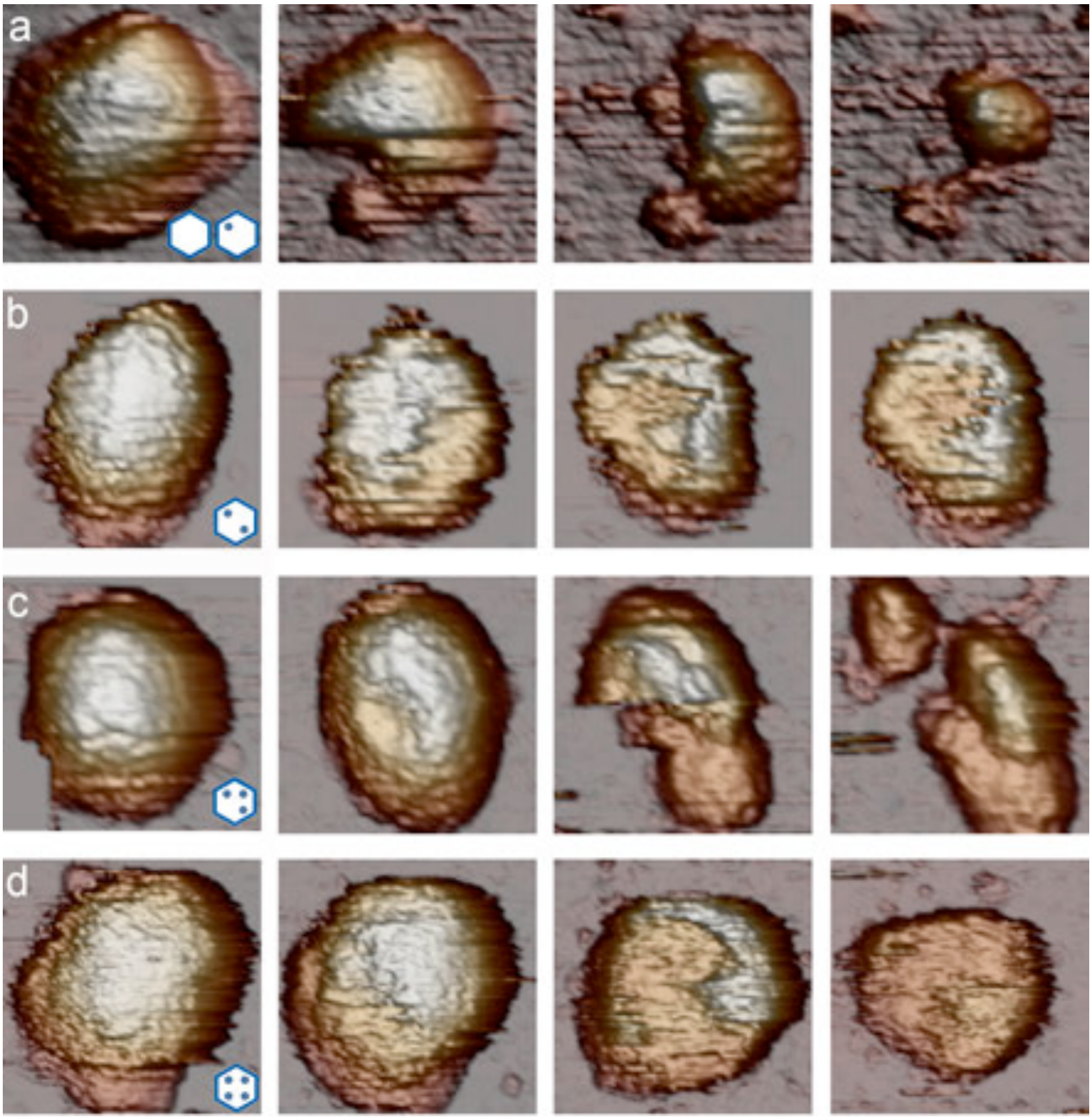

Supplementary Figure 4: AFM disruption images of E1-E6 IBDV particles by AFM tip mechanical fatigue. Selected succesive frames during capsid disruption of a (a) E1, (b) E3, (c) E4 and (d) E6 IBDV capsid. Observe that the RNP core is visible and stable in partially disrupted E3 and E6 particles. Cartoon of hexagons show the amount of RNP (dots) packed inside the particle for each type of IBDV population (first frame, bottom, right).
